# Optimization of Pressurized Liquid Extraction of Three Major Acetophenones from *Cynanchum bungei* Using a Box-Behnken Design

**DOI:** 10.3390/ijms131114533

**Published:** 2012-11-08

**Authors:** Wei Li, Li-Chun Zhao, Yin-Shi Sun, Feng-Jie Lei, Zi Wang, Xiong-Bin Gui, Hui Wang

**Affiliations:** 1 College of Chinese Medicinal Materials, Jilin Agricultural University, Changchun 130118, China; E-Mails: liwei7727@126.com (W.L.); Fengjie_lei@163.com (F.-J.L.); wangzi8020@126.com (Z.W.); 2 The Affiliated Ruikang Hospital, Guangxi University of Chinese Medicine, Nanning 530011, China; E-Mail: hyzlc@126.com; 3 College of Agronomy, Shandong Agricultural University, Taian 271018, China; E-Mail: sunyinshi2002@163.com; 4 China-Japan Union Hospital, Jilin University, Changchun 130033, China

**Keywords:** pressurized liquid extraction, acetophenones, *Cynanchum bungei* Decne, Box-Behnken design, response surface methodology

## Abstract

In this work, pressurized liquid extraction (PLE) of three acetophenones (4-hydroxyacetophenone, baishouwubenzophenone, and 2,4-dihydroxyacetophenone) from *Cynanchum bungei* (ACB) were investigated. The optimal conditions for extraction of ACB were obtained using a Box-Behnken design, consisting of 17 experimental points, as follows: Ethanol (100%) as the extraction solvent at a temperature of 120 °C and an extraction pressure of 1500 psi, using one extraction cycle with a static extraction time of 17 min. The extracted samples were analyzed by high-performance liquid chromatography using an UV detector. Under this optimal condition, the experimental values agreed with the predicted values by analysis of variance. The ACB extraction yield with optimal PLE was higher than that obtained by soxhlet extraction and heat-reflux extraction methods. The results suggest that the PLE method provides a good alternative for acetophenone extraction.

## 1. Introduction

**Figure 1:**
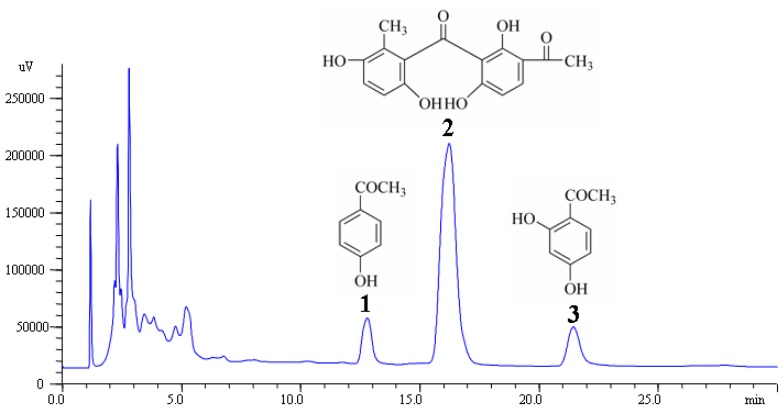
Chemical structures of 4-hydroxyacetophenone (**1**), baishouwubenzophenone (**2**), and 2,4-dihydroxyacetophenone (**3**) in *C. bungei*, and a high-performance liquid chromatogram of an extract of the three acetophenones.

The tuberous root of *Cynanchum bungei* Decne (Asclepiadaceae, ACB) has been traditionally used as one of the “four famous medicines” of Mount Tai. Modern pharmacological studies have shown it to have a wide range of pharmacological activities, such as anti-aging [1], gastroprotection [2], anti-tumor [3,4], immunoregulation [5] and tyrosinase inhibition [6]. Previous reports have found that acetophenones, primarily 4-hydroxyacetophenone (1), baishouwubenzophenone (2) and 2,4-dihydroxyacetophenone (3) are the major secondary metabolites of *C. bungei*[7,8] (Figure 1).

Natural product extraction forms the basis of the growing medicinal chemistry industry. Classical extraction methods, such as soxhlet extraction (SE) and heat-reflux extraction (HRE), are both time and solvent consuming [9]. Therefore, it is of considerable interest to find a better extraction method for the natural product industry. During the last decade, new extraction techniques emerged that will certainly supersede traditional extraction technologies in the future. These include supercritical fluid extraction [10], pressurized liquid extraction (PLE) [11], ultrasound-assisted extraction [12], and microwave-assisted solvent extraction [13]. Among these extraction technologies, pressurized liquid extraction (PLE), although not a very new technology for extraction, offers many significant advantages, including the requirement for only small volumes of solvents, the possibility for automation, and faster extraction than classical methods [14,15]. PLE is carried out under pressure to maintain the solvent in its liquid state at high temperature [16,17]. High temperatures and pressures increase the penetration of solvent into the plant material and improve constituent solubilization, enhancing both extraction speed and yield [18]. In the last decades, many applications of PLE have been reported, such as the extraction of chromones [16], saponins [19], fatty acids [20,21], and capsaicinoids [22]. In the present investigation, PLE was applied to the extraction of the three main acetophenones from ACB to investigate the effects of high temperature and pressure on the efficiency of the extraction. Moreover, the extraction parameters, including extraction time, extraction temperature, and ethanol concentration, were optimized with a Box-Behnken design (BBD). The relationship between the extraction conditions and the extraction yield of ACB was studied by running an experimental design, constructing a mathematical model, and investigating the relationship by response surface methodology.

The main objective of this paper was to develop a rapid and simple PLE method for the extraction of the three main acetophenones of ACB. The parameters affecting extraction yields that is, extraction solvent, extraction temperature and extraction time were investigated in detail. The results indicate that PLE allows the extraction of acetophenones within a short time with a considerable extraction yield in comparison with other methods.

## 2. Results and Discussion

### 2.1. Selection of Variables

The selection of a suitable extraction solvent is a key step for extracting natural compounds using PLE. Because various mixtures of ethanol and water have been applied for the conventional extraction of acetophenones [7,8], the effect of ethanol concentration on extraction yield of acetophenones was evaluated. Taking into account the influence of higher solubility and diffusion rate at higher temperatures [23], the effect of temperature on acetophenone extraction was investigated from 100 to 180 °C. As mentioned above, extraction time could be reduced in comparison with the conventional method, at high temperatures and a pressure enhanced extraction speed. Thus, in the present study, three points of extraction time from 8 to 20 min were chosen. Generally, pressure did not significantly influence extraction efficiency during the extraction of plant material. Based on the effects of pressure within the range permitted and results from the literature, a default level of 1500 psi was selected [16,17]. In addition, preliminary experiments showed that acetophenone extraction yields increased slowly without significant differences after one, two, or three extraction cycles (data not shown). One extraction cycle was estimated as sufficient to enable the majority of acetophenones to be released into the solvent [24]. The flush volume was set at a default value of 60%.

### 2.2. Model Fitting

To obtain a more realistic model, it was necessary to investigate the process variables. Preliminary trials enabled a range of ethanol concentrations (*X*_1_, 30%–100%), extraction times (*X*_2_, 8–20 min), and extraction temperatures (*X*_3_, 100–180 °C) to be tested. The entire design consisted of 17 experimental points as listed in Table 1, and five replicates (runs 13–17) at the center of the design were used to estimate a pure error sum of squares. The experiments were performed in triplicate at all design points in a random order.

As Table 2 shows, an analysis of variance (ANOVA) of extraction yield of acetophenones indicated that the experimental data had a determination coefficient (*R*^2^) of 0.9971 with the calculated model and no significant lack of fit at *p* > 0.1. This finding means that the calculated model was able to explain 99.71% of the results. The results indicated that the model used to fit response variables was significant (*p* < 0.0001) and adequate to represent the relationship between the response and the independent variables [25]. An *F* test suggested that the model had a very high model *F*-value (*F* = 83.52), indicating that this model was highly significant. The *R*^2^_adj_ (adjusted determination coefficient), which is the correlation measure for testing the goodness-of-fit of the regression equation [26], was 0.9789, which indicated that only 2.11% of the total variations were not explained by the model. Meanwhile, a relatively lower value of coefficient of variation (CV = 1.10) showed better precision and reliability of the experiments carried out [27,28].

Table 3 shows that the acetophenone extraction yield was affected most significantly by ethanol concentration (*X*_1_) and static extraction time (*X*_2_) (*p* < 0.001), followed by extraction temperature (*X_3_*) (*p* < 0.005). It was evident that two quadratic parameters (*X*_2_^2^, *X*_3_^2^ ) and two interaction parameters (*X*_1_*X*_2_, *X*_2_*X*_3_) were significant at the level of *p* < 0.0001. The predicted response *Y* for the yield of acetophenones could be expressed by the following second-order polynomial equation in terms of coded values:

(1) $$Y=856.7+25.64X_{1}-26.53X_{2}-24.06X_{3}+65.25X_{1}X_{2}+8.78X_{1}X_{3}-36.50X_{2}X_{3}-21.56X_{1}^{2}-50.79X_{2}^{2}-37.96X_{3}^{2}$$

where *Y* is the yield of the three acetophenones (μg/g), and *X*_1_, *X*_2_, and *X*_3_ are the coded variables for ethanol concentration, static extraction time, and extraction temperature, respectively.

### 2.3. Analysis of Response Surface

**Figure 2:**
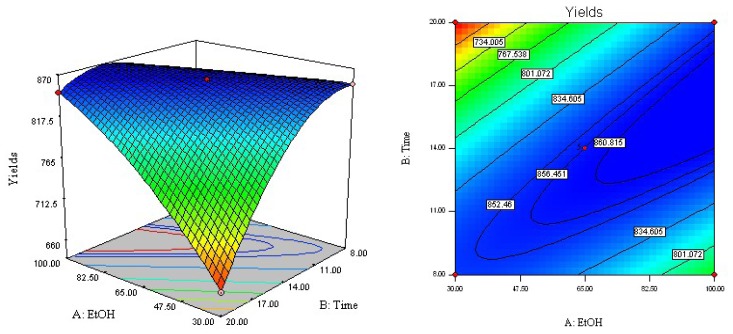
Response surface and contour plots of ethanol concentration and extraction time.

**Figure 3:**
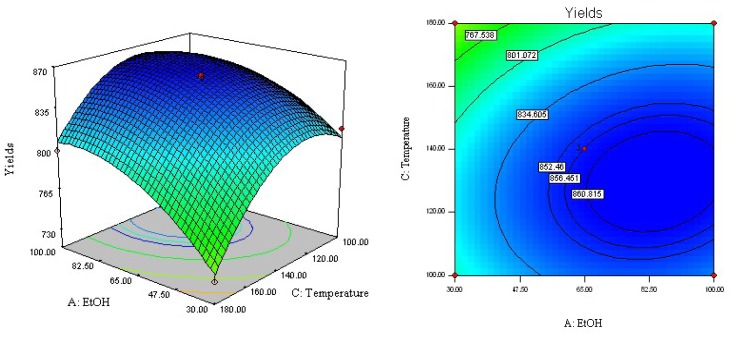
Response surface and contour plots of ethanol concentration and extraction temperature.

**Figure 4:**
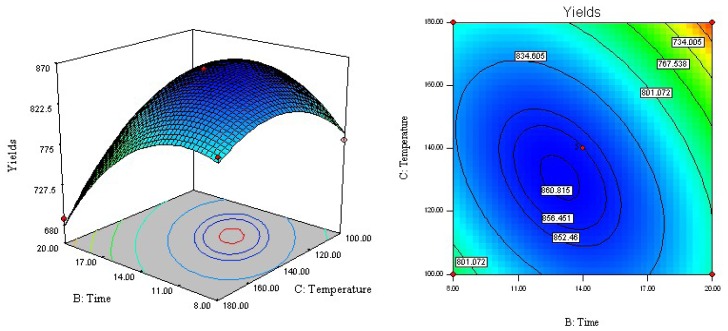
Response surface and contour plots of extraction temperature and extraction time.

The regression equation was graphically represented by three-dimensional response surface and two-dimensional contour plots. To visualize three-dimensional response surfaces, the response model was obtained against two experimental factors, whereas the third was held constant at its optimum value. From the three-dimensional response surface curves and contour plots shown in Figures 2–4, the effects of the independent variables and their mutual interaction on the yield of acetophenones in ACB can be seen.

Figure 2 shows the interaction between ethanol concentration (*X*_1_) and static extraction time (*X*_2_) with a fixed extraction temperature of 140 °C on the yield of acetophenones in ACB. An increase in ethanol concentration from 30% to 65% with a static extraction time from 8 to 14 min resulted in a maximum acetophenone yield of 858.8 μg/g. With increases of ethanol concentration over 65%, there was a gradual decline in the response, and static extraction times over 14 min did not show any obvious effect on extraction yield. These findings could be explained by the fact that increasing static extraction time may accelerate the chemical decomposition of bioactive compounds during the extraction process, which may result in a lower extraction yield. In other words, the yield of acetophenones could reach a peak value (858.8 μg/g) with an ethanol concentration of 65% and a static extraction time of 14 min.

The effect of ethanol concentration (*X*_1_) and extraction temperature (*X*_3_) on the yield of acetophenones in ACB is shown in Figure 3. When the extraction time was fixed at 14 min, the highest yield of acetophenones reached 860.3 μg/g under conditions of 140 °C and 65% ethanol. It can be also observed that increasing ethanol concentration from 30% to 65% and increasing extraction temperature from 100 to 140 °C increases the extraction yield of target compounds. However, the effects of extraction temperature on acetophenone yield were weaker than those of ethanol concentration. Moreover, there were no significant synergistic effects of ethanol concentration with extraction temperature on the yield of acetophenones (*p* = 0.088).

Figure 4 shows the response surface function developed by the model for extraction time (*X*_2_) and temperature (*X*_3_) with a fixed ethanol concentration of 65%, giving the deduced response of 857.8 μg/g at 14 min and 140 °C. ANOVA also indicated that the interaction of static extraction time and extraction temperature had a very significant effect on extraction yield (*p* < 0.0001), such that the highest extraction yield could be achieved when using an extraction temperature of 120 °C and a static extraction time of 17 min. In other words, a temperature increase was accompanied by an increase in extraction yield until reaching 120 °C. However, the yield of acetophenones decreased at temperatures greater than 120 °C. This finding can be explained, at least in part, by the non-selective extraction of various compounds and/or the degradation of acetophenones at high temperature [23].

### 2.4. Optimization of Extraction Parameters and Validation of the Model

In this study, the aim of the optimization was to find conditions that gave the maximum extraction yield of the three main acetophenones of ACB. Software (Design Expert 7.1.6) predicted the optimum ethanol concentration, static extraction time, and extraction temperature as 100%, 17.29 min, and 121.39 °C, respectively. The software also predicted the extraction yield of total acetophenones as 874.9 μg/g.

As shown in Table 4, five parallel experiments were carried out under the optimal conditions of ethanol concentration of 100%, a static extraction time of 17 min, and an extraction temperature of 120 °C, and the average extraction yield of total acetophenones was 877.8 μg/g. Compared with the value predicted by Design Expert 7.1.6 (Cisco Systems, Inc., USA), the results showed that the predicted value was very close to the actual results, indicating that the optimization was reliable in the present study.

### 2.5. Comparison of Different Extraction Techniques

**Figure 5:**
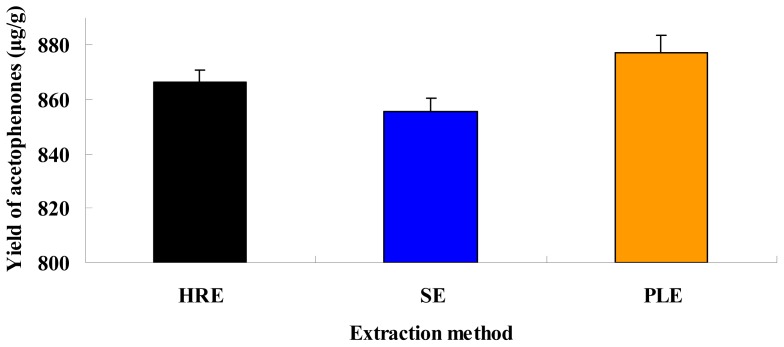
Extraction yields of acetophenones with different extraction methods.

In the present study, different extraction techniques including PLE, SE, and HRE were compared for acetophenone yield. The extraction yields of the three acetophenones obtained by the three extraction methods under optimal conditions are shown in Figure 5. The extraction yields of total acetophenones by PLE, HRE, and SE were 877.4, 866.6, and 855.6 μg/g, respectively. The extraction yields of 4-hydroxyacetophenone, baishouwubenzophenone, and 2,4-dihydroxyacetophenone by PLE were 46.2, 344.5, and 486.7 μg/g, respectively.

The static extraction time of PLE, SE and HRE were 17 min, 6 h and 9 h, respectively. The extraction yields of acetophenones obtained using PLE were higher than those using the SE and HRE methods. The modified PLE method was complete in only 17 min at 120 °C, whereas completing the SE and HRE methods depended to a large extent on the extraction time and number of cycles. As for the PLE method, the target compounds quickly swelled at high temperatures with aqueous ethanol, the extraction time was reduced significantly, and extraction efficiency was considerably increased. Therefore, PLE represents a simple and efficient technique for the extraction of acetophenones in ACB.

### 2.6. Validation of the HPLC Method

The proposed analytical high-performance liquid chromatography (HPLC) method was validated for various parameters, such as linear range, precision, and recovery. Calibration curves were carried out with six concentrations of a standard solution. Intra- and inter-day precision were also employed. The relative standard deviation (R.S.D.) values of the peak areas for the three flavones were less than 2.2%. The recovery of the acetophenones ranged from 99.2% to 101.1%, and their R.S.D values were less than 1.2%.

## 3. Experimental

### 3.1. Plant Material

The tuberous roots of ACB were kindly supplied by the Medicinal Plant Farm of Shandong Agricultural University and identified by Dr. Yin-shi Sun of the College of Agronomy, Shandong Agricultural University. The cut pieces were ground to obtain a relatively homogenous powder (60–80 mesh). The powder was dried at 60 °C and blended before use.

### 3.2. Chemicals and Reagents

Methanol was of HPLC grade from Fisher Chemicals (USA). Other chemicals, such as ethanol and CCl_4_, were all analytical grade from Beijing Chemical Factory. Water was purified using a Milli-Q water purification system (Milipore, USA). Standards of 4-hydroxyacetophenone, baishouwubenzophenone, and 2,4-dihydroxyacetophenone were kindly supplied by Dr. Yinshi Sun of Shandong Agricultural University.

### 3.3. Pressurized Liquid Extraction

PLE was performed with a Dionex ASE 300™ instrument equipped with a solvent controller (Dionex, Sunnyvale, CA, USA). The system consisted of a high-pressure pneumatic solvent pump capable of 1500 psi at elevated flow rate; an extraction solvent-pressurized bottle; a carousel for 12 extraction cells (100 mL each); a carousel for 250-mL collection vials; a microprocessor for storing and editing parameters, such as temperature, time, and pressure; and infrared sensors to detect the arrival of the fluid in the collection vial and to monitor fluid levels during extract collection. Since the flush volume was set at a default value of 60%, approximately 160 mL solvent were used per extraction cycle. In the present investigation, 100-mL extraction cells were used and filled with 5.0 g sample powder.

### 3.4. HRE

The sample powders (1.0 g) were weighed and placed into a round-bottomed flask with 50 mL methanol solution; the flask was placed into a water bath, connected to cooling water, and allowed to reflux for 2 h under a temperature of 80 °C. This process was repeated three times, and the total extraction time was 6 h.

### 3.5. SE

The sample powders (1.0 g) were placed into a soxhlet apparatus and extracted with 50 mL methanol for 6 h at a temperature of 80 °C.

After the extraction procedure, the filtered solutions were concentrated to dryness under vacuum at 40 °C. The obtained dry extracts were diluted in 10 mL methanol, and the supernatant was filtered through a 0.22-μm nylon membrane and analyzed by HPLC. Three replicate injections were analyzed to determine the extraction yields of acetophenones with a mean peak area.

### 3.6. HPLC Analysis of Acetophenones

The three acetophenones, 4-hydroxyacetophenone (**1**), baishouwubenzophenone (**2**), and 2,4-dihydroxyacetophenone (**3**), were quantified by HPLC coupled with UV detection (HPLC-UV). HPLC analyses were performed with an HPLC instrument (Agilent 1100, USA) equipped with a quaternary solvent delivery system, a column oven, and an UV detector (Agilent 1100, VWD, USA). Twenty microliters of sample solution were injected into the column manually. Separation was achieved on an Hypersil ODS2 column (4.6 mm × 250 mm, 5 μm) from Dalian Elite Analytical Instruments Co., Ltd. The column temperature was set to 25 °C and the detection wavelength was set at 280 nm. The mobile phase was 25% methanol with isocratic elution at a flow rate of 1.0 mL/min. The amount of each acetophenone was expressed in terms of microgram per gram dry weight of sample material. Figure 1 shows a chromatogram for the simultaneous determination of the three main acetophenones of ACB.

### 3.7. Response Surface Modeling

A central composite design (BBD, Design Expert software, Trial Version 7.1.6) with three variables was used to determine the response pattern and establish a model. The three variables used in this study were ethanol concentration (*X*_1_), static extraction time (*X*_2_), and temperature (*X*_3_) with three levels (−1, 0, 1) for each variable, whereas the dependent variable was acetophenone yield. The proper range of the three variables was determined by a single-factor experiment for acetophenone production.

Experimental data were fitted to a quadratic polynomial model and a regression coefficient was obtained. The non-linear, computer-generated, quadratic model used in the response surface was as follows:

(2) $$Y=\beta_{0}+\sum_{j=1}^{k}\beta_{j}X_{j}+\sum_{j=1}^{k}\beta_{jj}X_{j}^{2}+\sum \sum_{i<j}\beta_{ij}X_{i}Y_{j}$$

where *Y* is the estimated response, *β_0_*, *β_j_*, *β_jj_*_,_ and *β_ij_* are the regression coefficients for intercept, linearity, square, and interaction, respectively, and *X_i_* and *X_j_* are the independent coded variables.

### 3.8. Data Analysis

All data presented are the means ± SD of three determinations. Stat-Ease Design-Expert 7.1.6 software (Trial Version, Stat-Ease Inc., Minneapolis, MN, USA) was used for the experimental design, data analysis, and model building. The quality of the fit of the polynomial model equation as expressed by the coefficients was checked by *F* test and *p*-value.

## 4. Conclusions

An adequate quadratic polynomial model for predicting acetophenone yield from ACB using PLE was determined according to an optimized design. ANOVA analysis indicated that three parameters—extraction time, temperature and ethanol concentration—significantly influenced extraction efficiency. The optimal condition derived using a BBD was determined to be 17 min, 120 °C and 100% ethanol. Under optimal conditions, the experimental value agreed with the predicted value by ANOVA. Compared with the conventional extraction method, the optimized PLE method provided higher extraction efficiency in a shorter time with low solvent consumption.

## Figures and Tables

**Table 1:** A Box-Behnken experimental design with independent variables.

Run	Coded variables levels			Yield of acetophenones (μg/g)	
	
	*X*_1_, ethanol (%)	*X*_2_, time (min)	*X*_3_, temperature (°C)	Actual values	Predicted values
1	30	8	140	850.4	850.5
2	100	8	140	772.8	771.3
3	30	20	140	665.4	666.9
4	100	20	140	848.8	848.7
5	30	14	100	812.3	804.4
6	100	14	100	844.4	838.1
7	30	14	180	732.4	738.7
8	100	14	180	799.6	807.5
9	65	8	100	774.2	782.0
10	65	20	100	795.6	802.0
11	65	8	180	813.3	806.9
12	65	20	180	688.7	680.9
13	65	14	140	846.5	856.7
14	65	14	140	857.8	856.7
15	65	14	140	858.5	856.7
16	65	14	140	860.3	856.7
17	65	14	140	860.4	856.7

**Table 2:** Analysis of variance for the fitted quadratic polynomial model of extraction of acetophenones.

Source	SS	DF	MS	*F*-value	*p*-value	
Model	58991.25	9	6554.58	83.52	<0.0001	significant
Residual	549.33	7	78.48			
Lack of fit	414.19	3	138.06	4.09	0.1037	insignificant
Experimental error	135.14	4	33.79			

SS, sum of squares; DF, degrees of freedom; MS, mean square.

**Table 3:** Estimated regression model of the relationship between response variables (yield of the three acetophenones) and independent variables (*X*_1_, *X*_2_, and *X*_3_).

Variables	DF	SS	MS	*F*-values	*p*-value
*X*_1_	1	5258.25	5258.25	67.01	<0.0001
*X*_2_	1	5628.61	5628.61	71.72	<0.0001
*X*_3_	1	4632.03	4632.03	59.03	0.0001
*X*_1_*X*_2_	1	17030.25	17030.25	217.01	<0.0001
*X*_1_*X*_3_	1	308.00	308.00	3.92	0.0880
*X*_2_*X*_3_	1	5329.00	5329.00	67.90	<0.0001
*X_1_^2^*	1	1957.65	1957.65	24.95	0.0016
*X_2_^2^*	1	10860.51	10860.51	138.39	<0.0001
*X_3_^2^*	1	6068.01	6068.01	77.32	<0.0001

**Table 4:** Optimum conditions and the predicted and experimental values of response under optimum conditions.

	Ethanol (%)	Extraction time (min)	Temperature (°C)	Yield of acetophenones (μg/g)
Optimum conditions	100	17.29	121.39	874.9 (predicted)
Modified conditions	100	17.00	120.00	877.8 ± 21.11 (actual)
